# Short tandem repeat expansions in 1532 patients with neurodegenerative dementia in China

**DOI:** 10.1002/alz70855_101521

**Published:** 2025-12-23

**Authors:** Yuan Zhu, Xuewen Xiao, Tianyan Xu, Qijie Yang, Xiaoli Hao, Sizhe Zhang, Yun Tian, Bin Jiao, Lu Shen

**Affiliations:** ^1^ Xiangya Hospital, Central South University, Changsha, Hunan, China

## Abstract

**Background:**

Pathogenic short tandem repeat (STR) expansions cause many neurodegenerative diseases, including frontotemporal dementia (FTD), Huntington's Disease (HD) and neuronal intranuclear inclusion disease (NIID). These STR‐associated neurodegenerative dementias are characterized by phenotypic and genetic heterogeneity. However, reports on STRs in large neurodegenerative dementia cohort in China are lacking.

**Method:**

To determine the contribution of STRs in neurodegenerative dementias, we used ExpansionHunter (EH) and ExpansionHunterDenovo (EHDn) to assess 19 neurodegenerative disease‐associated STRs in whole‐genome sequencing data from 928 patients with AD, 231 patients with PSP, 208 patients with FTD, and 165 patients with DLB. We completed polymerase chain reaction (PCR) validation for EH calls that exceeds the intermediate thresholds of STR. For EHDn calls, we performed PCR tests for CCGGGG expansions with high counts in motif‐based comparison in FTD cohort.

**Result:**

In the FTD cohort, we identified 15 pathogenic expansions in *C9orf72* (12 cases, 10 EH calls+2 EHDn calls), *HTT* (1 case), and *Notch2NLC* (2 cases). The AD cohort validated 6 pathogenic expansions in *C9orf72* (2 cases), *HTT* (1 case), *Notch2NLC* (2 cases), and *FMR1* (2 cases). *PPP2R2B* (1 case) and *AR* (1 case) expansions were found in the DLB cohort. 12.5% of clinically diagnosed FTD cases had at least one expanded STR allele above the intermediate threshold, followed by 7.36% of PSP cases. In the PSP cohort, 2.6% carried a *TBP* intermediate expanded allele, which was much higher than other subgroups. While 1.82% of DLB cases carried a HTT intermediate expansion.

**Conclusion:**

Our findings suggest that the clinical and pathological pleiotropy of neurodegenerative disease genes and highlight their importance in neurodegenerative dementias.

